# High Incidence of Zika or Chikungunya Infection among Pregnant Women Hospitalized Due to Obstetrical Complications in Northeastern Brazil—Implications for Laboratory Screening in Arbovirus Endemic Area

**DOI:** 10.3390/v13050744

**Published:** 2021-04-23

**Authors:** Iracema J. A. A. Jacques, Leila Katz, Marília A. Sena, Ana B. G. Guimarães, Yasmim L. Silva, Gabriela D. M. Albuquerque, Raisa O. Pereira, Camila A. M. C. de Albuquerque, Maria Almerice L. Silva, Paula A. S. Oliveira, Maria de Fátima P. M. Albuquerque, Marli T. Cordeiro, Ernesto T. A. Marques, Rafael F. O. França, Celina M. T. Martelli, Priscila M. S. Castanha, Cynthia Braga

**Affiliations:** 1 Instituto Aggeu Magalhães, Fundação Oswaldo Cruz Pernambuco, Recife CEP 50740-465, PE, Brazil; iracema__alves@hotmail.com (I.J.A.A.J.); mariliasena97@hotmail.com (M.A.S.); anabgiles@gmail.com (A.B.G.G.); yasmimlucena17@gmail.com (Y.L.S.); almerice.lopes@fiocruz.br (M.A.L.S.); oliveiraaspaula@gmail.com (P.A.S.O.); militaofatima@gmail.com (M.d.F.P.M.A.); marli.tenorio@gmail.com (M.T.C.); rafael.franca@fiocruz.br (R.F.O.F.); turchicm@gmail.com (C.M.T.M.); 2 Instituto de Medicina Integral Prof. Fernando Figueira, Recife CEP 50070-550, PE, Brazil; katzleila@gmail.com (L.K.); gabi.dmilitao@gmail.com (G.D.M.A.); oliveira.raisa@yahoo.com.br (R.O.P.); camila.med2016@gmail.com (C.A.M.C.d.A.); 3 Department of Infectious Diseases and Microbiology, University of Pittsburgh, Pittsburgh, PA 15261, USA; emarques@cpqam.fiocruz.br (E.T.A.M.J.); castanha.priscila@gmail.com (P.M.S.C.); 4 Faculdade de Ciências Médicas, Universidade de Pernambuco, Recife CEP 50100-130, PE, Brazil

**Keywords:** Zika virus, dengue virus, chikungunya virus, pregnancy complications

## Abstract

The diagnostic of arbovirus-related obstetric complications in high-risk pregnancy and childbirth care is challenging, especially in endemic areas. We conducted a prospective study to track active or recent Zika (ZIKV), dengue (DENV), or chikungunya (CHIKV) virus infection among hospitalized pregnant women (PW) with obstetric complications in a hospital at the epicenter of Zika outbreak and ZIKV-related microcephaly in Brazil. Clinical data and blood samples were collected at enrollment and 10 days after the admission of study participants, between October 2018 and May 2019. Further clinical data were extracted from medical records. Samples were screened by molecular and serological tests. Out of 780 participants, 93.1% (95% CI: 91.1–94.7%) presented previous DENV exposure (IgG). ZIKV, CHIKV, and/or DENV laboratory markers of recent or active infection were detected in 130 PW, yielding a prevalence of 16.6% (95% CI: 14.2–19.5%); 9.4% (95% CI: 7.4–11.7%), 7.4% (95% CI: 5.7–9.7%), and 0.38% (95% CI: 0.1–1.2%) of CHIKV, ZIKV, and DENV infections, respectively. Most ZIKV infections were detected by molecular assays (89.6%), while CHIKV infections were detected by serology (95.9%). Our findings highlight the need for arbovirus infections screening in PW with obstetrical complications, potentially associated to these infections in endemic areas regardless of the signs or symptoms suggestive of arboviral disease.

## 1. Introduction

Vector-borne diseases, especially arboviruses, account for more than 17% of all infectious diseases worldwide and they are responsible for more than 1 million deaths annually [1]. It is estimated that half of the world population is at risk of being infected by the dengue virus (DENV, genus *Flavivirus*, family *Flaviviridae*) every year [1,2]. The recent circulation of Zika virus (ZIKV, genus *Flavivirus*, family *Flaviviridae*) in the Pacific region, the Americas, Africa, and Southeast Asia has led to an unprecedented increase in disease-related complications, including Guillain–Barré syndrome and congenital Zika syndrome [3]. In recent decades chikungunya virus (CHIKV, genus *Alphavirus*, family *Togaviridae*) has become the focus of attention, due to its rapid spread and the high magnitude of outbreaks in Africa, Asia, Indian Ocean islands, and Europe, and more recently in the Americas, where more than two million suspected cases have been recorded [4,5].

Nearly 90% of the annual pregnancies worldwide occur in endemic or epidemic areas of arboviruses (including ZIKV, DENV, and CHIKV) and the remaining 10% of pregnant women (PW) are at risk of exposure while traveling to areas of arbovirus circulation [6]. The clinical manifestations of arboviral diseases in PW are generally similar to those observed in the general population. However, this population have greater chance of developing severe clinical forms, as well as a higher risk for obstetric complications, namely, premature birth, placental abruption, pre-eclampsia, vaginal bleeding, and disease-related deaths [7,8,9]. Furthermore, mother-to-child arbovirus transmission via the placenta or during childbirth may cause severe neonatal infection [6,7,8,9]. 

The clinical and laboratorial diagnostic of arbovirus-related obstetric complications is challenging, especially in endemic areas. A considerable part of the PW infected with arbovirus are asymptomatic or manifest mild forms of the disease with nonspecific symptoms [6,10,11], making it difficult to distinguish the obstetric complications related to arboviral infections from those due to other causes. Moreover, the accuracy of the available diagnostic tests in areas of co-circulation of multiple arboviruses is limited [12,13], and knowledge on the kinetics of the laboratory markers of arboviral infections during pregnancy is still scarce [14,15,16]. 

The Northeast region of Brazil has been affected by successive waves of severe arboviruses outbreaks since the introduction of DENV in the 1980’s [17]. This region has registered the second highest incidence of dengue and it is responsible for around a quarter of the deaths by dengue infection in the country [18]. Following the emergence of ZIKV in Brazil, the region concentrated about 70% of the cases of ZIKV-related congenital microcephaly [19]. The Northeast region also accounted for most reported cases of chikungunya during the epidemic that occurred after the introduction of CHIKV in Brazil in 2014 [20,21].

A previous study conducted during the peak or decline of the first Zika epidemic in Brazil reported around 70% of previous ZIKV infection among PW [22,23]. However, information on the frequency of arbovirus-related obstetric complications in high-risk pregnancy and childbirth care services in these areas are still scarce. Therefore, we carried out a prospective study to estimate the frequency of laboratory markers of active or recent infection of ZIKV, DENV, or CHIKV among PW hospitalized due to obstetric complications in a reference maternity hospital in this region. The study was also designed to investigate clinical and laboratorial features of arbovirus-related obstetric complications in this population. Here, we describe the frequency of laboratory markers of these infections and the signs and symptoms of arboviral disease reported by this population. We also compared the observed pattern of occurrence of these infections among the PW in relation to the data reported by the national arbovirus disease surveillance system for the same setting.

## 2. Materials and Methods 

### 2.1. Study Design and Population

The prospective study was carried out at the maternity ward of the Instituto de Medicina Integral Prof. Fernando Figueira (IMIP), a publicly funded reference hospital for assistance of high-risk pregnancy and childbirth, located in the city of Recife, the capital of Pernambuco state, Northeast region of Brazil. The city of Recife is a hyperendemic area of arbovirus transmission and it was considered the epicenter of outbreaks of Zika disease [24] and ZIKV-related microcephaly in Brazil [25]. During the study recruitment (October 2018 and May 2019), 8705 cases of dengue, 160 of Zika, and 675 of chikungunya were reported in the general population by the local surveillance system [26]. Moreover, a population-based serological survey conducted by our group in a similar period (August 2018 to February 2019) have found the prevalence of serological markers of recent ZIKV and CHIKV infection (IgM antibodies) of 1.2% (95% CI: 0.6–2.2%) and 4.4% (95% CI: 3.2–6.1%), respectively, in the women at the reproductive age (15–49 years old, *n* = 855) living in this setting (data not published).

We consecutively enrolled PW with gestational age ≥ 27 weeks, aged ≥ 15 years, resident in the metropolitan area of Recife, and who were admitted to the maternity ward due to obstetric complications, regardless of having reported signs or symptoms of arboviral disease during the current pregnancy. Screening for DENV, CHIKV, or ZIKV infections are not included in the routine exams of the health unit, unless requested by the attending physician. Obstetric complications included were obstetrical bleeding, placental abruption, premature labor, premature amniorrhexis, chorioamnionitis, oligohydramnios, gestational diabetes, gestational hypertension, pre-eclampsia or eclampsia, and HELLP syndrome. We excluded those PW diagnosed with cancer, diabetes mellitus and chronic hypertension before the current pregnancy, HIV infection, syphilis, TORCH (Toxoplasmosis, Syphilis, Varicella Zoster, Rubella, Cytomegalovirus and Herpes simplex virus), autoimmune diseases, decompensated chronic heart disease, hemophilia A and B, Von Willebrand disease, and continued use of antiepileptic drugs.

### 2.2. Data Collection

The participant or their legal guardians (if under 18 years old or if not clinically able) were informed about the study goals, invited to participate, and asked to read and sign the informed consent form. All study participants had access to the results of the laboratory tests. The PW with molecular or serological markers of recent or acute ZIKV infection were immediately contacted by the research team and they were instructed to seek pediatric assistance for clinical evaluation of their babies. The protocol for this study was approved by the Research Ethics Committee of the Fiocruz Pernambuco (CAEE: 73121517.0.0000.5190) and IMIP (CAEE: 73121517.0.3001.5201).

At the time of enrollment, sociodemographic (age, self-reported race/skin color, schooling, and family income) and clinical data (parity, maternal complications in previous pregnancies, prenatal care, type of obstetric complication in current pregnancy, and signs and symptoms suggestive of arbovirus infection) were obtained using a standardized questionnaire. Moreover, a maternal blood sample was obtained for laboratory investigation of arbovirus infection. Further clinical data and results of laboratory tests and imaging exams performed during hospitalization were extracted from the patient’s medical records using a standardized form.

A second clinical assessment of the participants was performed 10 days after participant’s admission to the study for collecting further clinical information, including signs or symptoms of arboviral disease following the first interview, and an additional venous blood sample. The diagnosis of obstetric complications provided by the medical assistants during hospitalization were reviewed by the research team based on case definition criteria (clinical and laboratory), established in clinical guidelines [27,28,29,30,31,32,33,34,35,36].

### 2.3. Laboratory Procedures

All maternal samples were screened for molecular and serological detection of DENV, ZIKV, and CHIKV infections. All laboratory procedures were conducted at the Virology Department (LaViTE) of Fiocruz Pernambuco.

The detection of DENV, ZIKV, and CHIKV specific genomes was performed by quantitative real-time PCR (qRT-PCR). The presence of virus-specific RNA was first assessed in the PW samples collected at the time of enrollment. If positive, follow-up samples were also tested to assess viral RNA persistence. Viral RNA was extracted using the ReliaPrepTM Viral TNA Miniprep System (Promega Corporation, Madison, WI, USA) following the manufacturer’s instructions. The US Centers for Disease Control and Prevention (CDC) one-step, single reaction qRT-PCR Trioplex assay was performed for the concurrent detection of DENV, ZIKV, and CHIKV RNA in the PW samples [37]. Positive and undetermined results for any virus by the qRT-PCR Trioplex assay were further confirmed by an additional one-step qRT-PCR for the detection of DENV, ZIKV, or CHIKV RNA separately using primers and probes described by Magalhães and colleagues [21].

The detection of DENV and ZIKV IgM antibodies was conducted using the CDC IgM-capture ELISA following a protocol previously described [38]. All PW’s samples were tested in parallel with ZIKV and DENV antigens to account for any potential flavivirus cross-reactivity. Samples were tested in duplicate, and the results were calculated as a ratio of the average optical density (OD) value of the test sample (P) divided by the average OD value of the negative control (N). P/N values of <2.0 were considered negative; >3.0, positive; and 2.0–3.0, equivocal. Positive results for both ZIKV and DENV antigens were considered as positive for ZIKV if the ZIKV P/N ratio was at least twice the DENV P/N ratio [38]. The detection of chikungunya IgM antibodies was assessed in all PW samples using a commercial ELISA IgM kit (Euroimmun, Lübeck, SH, Germany), following the manufacturer’s protocol.

All PW samples with positive or inconclusive results for ZIKV, DENV, or CHIKV by IgM ELISA were tested by plaque reduction neutralization test (PRNT). Virus-specific neutralizing antibodies were assessed using a protocol described in detail elsewhere [14]. PRNT assays were performed using Vero cells and virus strains isolated in the study area: DENV-1 (BR-PE/97-42735), DENV-2 (BR-PE/95-3808), DENV-3 (BR-PE/02-95016), DENV-4 (BR-PE/12-008), ZIKV (BR-PE243/2015), and CHIKV (PB-302). The cutoff for positive PRNT was defined based on a 50% reduction in plaque counts (PRNT50) in the lowest serum dilution tested (1:20). Neutralizing antibody titers for positive samples were calculated using a four-parameter nonlinear regression. Paired samples were assayed in parallel and a four-fold rise in neutralizing antibody titers between the samples was indicative of an acute infection. In addition to the PRNT, positive or inconclusive results for CHIKV by IgM ELISA were also tested for anti-CHIKV IgG antibodies using a commercial ELISA IgG kit (Euroimmun, Lübeck, SH, Germany) following the manufacturer’s protocol. 

Previous DENV exposure (DENV-specific IgG) was assessed in all PW samples collected at enrollment using a commercial indirect IgG ELISA (Alere, Waltham, MA, USA) that was performed according to the manufacturer’s instructions. 

### 2.4. Case Definition of Arbovirus Infection

PW with a positive qRT-PCR result for any of the viruses (DENV, ZIKV, or CHIKV) using the Trioplex assay and further confirmed by the in-house virus-specific qRT-PCR were considered as having an active arboviral infection (viremia). IgM or IgG seroconversion (negative in the first sample and positive in the second sample) or a four-fold rise in virus-specific neutralizing antibody titers between paired samples was also indicative of an active arbovirus infection (serological markers of active infection). 

Patients were considered as having a recent arbovirus infection of ZIKV, DENV, or CHIKV if the virus-specific IgM assay was positive in the first or in the paired samples. ZIKV cases were further assayed through PRNT and were discarded if the PRNT results did not indicate a recent infection. Dual infections were defined as evidence of infection (concurrent or sequential) with more than one virus by PCR or IgM test.

We defined suspected cases of DENV, ZIKV, and CHIKV infection based on clinical criteria and according to World Health Organization (WHO) case definitions. Zika suspected cases were defined as the report of rash and/or fever and at least one of the following signs or symptoms: arthralgia, arthritis, or conjunctivitis [39]. Chikungunya suspected cases were defined as the report triad of fever, rash, and joint manifestations [40] while suspected dengue cases were defined as the report of fever with two of the following symptoms: severe headache, pain behind the eyes, muscle and joint pains, nausea, vomiting, swollen glands, and rash [41]. 

### 2.5. Data Analysis 

Data were managed using REDCap electronic data capture tools [42] hosted at Oswaldo Cruz Foundation (FIOCRUZ), Brazil. Data analysis was performed using Stata Program, version 15 (StataCorp., CollegeStation, TX, USA). Data were summarized and frequency distributions were displayed in tables and graphs. Descriptive statistics for categorical variables were presented as percentages, and continuous variables as mean ± standard deviation (SD). The frequency distribution of the sociodemographic characteristics, arboviral infections, and associated clinical manifestations, as well as the profile of laboratory markers according to the type of arboviral infection, were analyzed. The temporal distribution (per epidemiological week) of the arboviral infections in the study population was described in relation to the incidence of reported cases of arbovirus disease (dengue, Zika, and chikungunya) in the general population of the study site, according to the national surveillance system [26]. Differences in the frequency of suspected Zika and chikungunya cases and in the frequency of signs and symptoms suggestive of arbovirus disease between infected and non-infected PW were analyzed by the Pearson Chi-square test, with a 5% significance level.

## 3. Results

**Figure 1:**
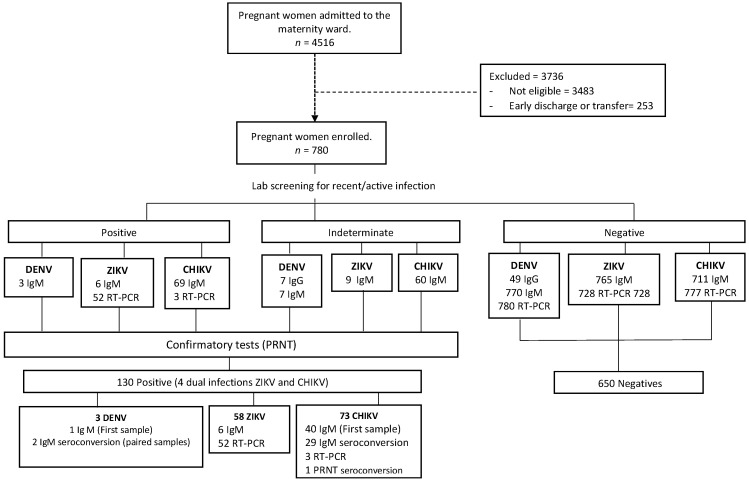
Flowchart of the study population.

From October 2018 to May 2019, 4516 PW were admitted to the maternity ward of IMIP. Out of 4263 (94.4%) women diagnosed with obstetric complications at the hospital admission, 806 (19%) met the inclusion criteria for this study. In total, 26 PW were excluded after clinical review by the research team and 780 were included in this study (Figure 1). Among the 780 participants enrolled, 728 (93.3%) completed the study protocol and had paired blood samples collected.

Table 1 shows the main sociodemographic and clinical characteristics of the participants. Pregnant women were mainly young (median age 26.5, range 15–47) and with self-reported mixed skin color (61.1%). Most participants had completed high school or university (48%) and they had a monthly family income of less than two minimum wages (82.8%). Gestational age ranged from 26 to 42 weeks (median 38), and most women had prenatal follow-up (97.2%) and a single pregnancy (91.3%). Most obstetric complications were hypertension disorders (60.1%), followed by premature labor or childbirth (14.1%), and gestational diabetes (12.6%) (Table 1).

Out of 780 PW screened, 16.6% (95% CI: 14.2–19.5%) had laboratory markers of recent or active arbovirus infection (ZIKV, CHIKV, and/or DENV); 9.4% (95% CI: 7.4–11.7%) CHIKV infections and 7.4% (95% CI: 5.7–9.7%) ZIKV infections. Only three PW (0.38%) had a recent DENV infection (95% CI: 0.1–1.2%) and 93.1% (95% CI: 91.1–94.7%) had previous exposure serological markers (anti-DENV IgG). 

**Figure 2:**
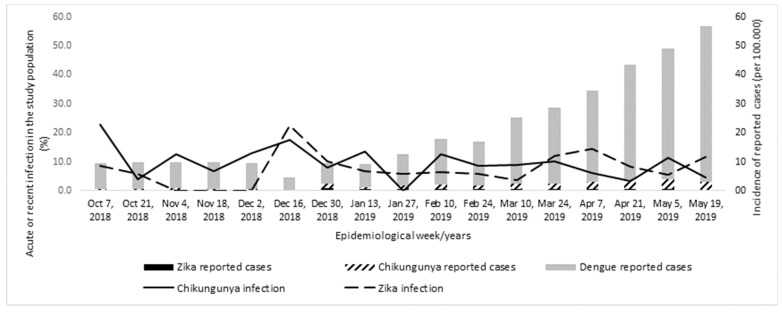
Temporal distribution of the ZIKV and CHIKV active or recent infection among screened pregnant women (lines) and incidence of arbovirus cases reported by the official surveillance system(bars) [26] from Recife, Pernambuco, October 2018 to May 2019.

Figure 2 shows the temporal distribution of the frequency of ZIKV and CHIKV infections in the study population and the incidence of reported cases of dengue, Zika, and chikungunya disease in the study setting, according to the passive surveillance system during the study period (from October 2018 to May 2019). The frequency of ZIKV and CHIKV infection among PW remained relatively constant over the period, although there was an increase in the incidence of reported cases of arboviruses, mainly dengue, from March 2019 (11th epidemiological week), in the general population of the study area, according to the official surveillance system. 

Among the 130 PW infected by arbovirus, 54 (41.5%) were infected by ZIKV, 3 (2.3%) by DENV, 69 (53.1%) by CHIKV and 4 (3.1%) had dual infections by CHIKV and ZIKV. Most cases (64.9%) had active infections (circulating viral RNA and/or seroconversion). ZIKV infections were mainly detected by molecular assays (89.6%), while CHIKV infections were mainly confirmed by serological tests (95.9%). All DENV infections (*n* = 3) were detected by seroconversion (Table 2).

A total of 10 participants were ZIKV qRT-PCR positives in the paired samples. Six of these participants had a time interval between samples collection higher than 15 days, indicating persistence of ZIKV viral RNA in the plasma. Among those, the period of persistence of viremia after delivery varied from 19 to 29 days (median = 25.6 days). One out of these six PW had reported symptoms suggestive of arbovirus (fever and myalgia) in the first trimester of gestation. 

Table 3 shows the frequency distribution of the suspected cases according to the WHO case definition, clinical signs and symptoms of arbovirus disease reported by PW and the main obstetrical complications according to the active/recent infection status (CHIKV or ZIKV). No statistically significant differences were found regarding the frequency of symptoms and obstetrical complications between arbovirus infected and uninfected PW. Less than 5% of those women infected were classified as suspected cases based on the WHO case definitions for Zika and chikungunya. Around 14% of the PW reported arthralgia, with a higher frequency of these symptoms being reported among the CHIKV infection, when compared to the ZIKV infected (18.8% versus 7.4%), although without statistically significant differences.

## 4. Discussion

This study was conducted two years after the Zika and chikungunya epidemic in a hyperendemic setting, considered the epicenter of the Zika epidemic in Brazil (24). We found that around 17% of the PW with obstetric complications had laboratory markers of active or recent arbovirus infections (CHIKV, ZIKV, or DENV) at the time of hospitalization. Recent CHIKV and ZIKV infection were the most frequent infections detected in this study population. Note that, the clinical suspicion of obstetric complication attributed to maternal arbovirus infection was not raised by the clinical staff in all cases, which were only identified after exhaustive laboratory investigation using different diagnostic methods.

At enrollment, 9.4% of the PW had laboratorial evidence of CHIKV infection, mainly detected by serological tests in one or in paired samples, while 7.4% of the PW had evidence of ZIKV infection, most detected by molecular assays. Recent DENV was the least frequent infection detected in this population, in line with the high prevalence of DENV-immune response found in our population (93.1%) and also with the results of other studies conducted in the same setting [14,16]. Surprisingly, the incidence of ZIKV infection of 7% observed in our study was similar to that observed in a prospective cohort study conducted in PW with obstetric complications during the peak of the epidemic in the city of Jundiai, Southeast region of Brazil [43]. These results call attention to the maintenance of high levels of exposure to arbovirus infection in PW in our region, three years after the occurrence of the Zika and chikungunya epidemics. 

The case frequency of arbovirus infection among PW, especially CHIKV and ZIKV, remained constant throughout the study period, confirming the co-circulation of the three viruses in the region and warning for the potential risk to the health of PW and their infants. This result is in accordance with that reported by the local surveillance system that show the co-circulation of the three viruses after two years of the epidemic in the study setting. However, contrary to what was observed in our study, most reported case incidence occurred at the expense of dengue instead of Zika and chikungunya. The incidence of reported cases, most confirmed by clinical and epidemiological criteria, suggests a possible underreporting of cases of Zika and chikungunya, due to the difficulty in differentiating these infections in areas of co-circulation of different viruses solely based on these criteria. These data demonstrate the limitations of using a passive surveillance system as a basis for investigation and for the clinical decision in the face of events potentially attributed to arbovirus infections in PW or newborns. This alert applies to both endemic and non-endemic countries, as reported in previous articles [44,45].

In our study, around 90% of the Zika infection are confirmed by the detection of viral RNA. Furthermore, out of ten ZIKV cases with positive qRT-PCR results in paired samples, six women showed evidence of viral RNA persistence in the plasma with a median detection of ZIKV RNA of 25 days. The viral RNA persistence found among our study population is in consonance with previous studies showing prolonged detection of ZIKV RNA in body fluids of symptomatic and asymptomatic ZIKV infected individuals, including PW [46,47]. Previous studies have reported a median time to loss of ZIKV RNA detection of around 10 and 14 days in plasma or serum samples of asymptomatic and symptomatic ZIKV infected patients, respectively [48]. More significantly, several case reports and prospective studies have demonstrated a longer time-to-loss of ZIKV RNA detection in samples of PW [46,49,50,51]. A prospective cohort study conducted in Puerto Rico reported a median detection of ZIKV RNA in serum around 3-fold longer among PW than non-pregnant women [46]. 

The prolonged ZIKV RNA detection in PW has been associated to the replication and persistence of ZIKV in placental trophoblasts and in the fetus that may act as a viral reservoir leading to the transfer of placenta or fetus-derived genetic material into the maternal circulation [52]. Moreover, changes in the immune response that occur in the distinct stages of gestation have also been suggested as a possible reason for the delayed viral clearance during pregnancy [53,54]. However, the mechanism underlying the longer time-to-loss of ZIKV RNA detection in PW and the persistence of RNA detection after delivery remain unknown. Further studies investigating whether this persistence is still the result of immunological changes in the postpartum period are needed to elucidate this question.

In contrast with the cases of ZIKV infection, nearly all CHIKV infection cases were confirmed by IgM and/or IgG seroconversion. Only three cases of CHIKV infections were confirmed by RNA viral detection. This result is consistent with a previous study analyzing ZIKV and CHIKV shedding and the kinetics of virus-specific antibodies in a cohort of Brazilian patients [55]. Bozza et al. demonstrated that although CHIKV viral loads are initially much higher than those observed in ZIKV infections, CHIKV RNA decline sharply to low levels following infection. The kinetics of IgM antibodies also differed between ZIKV and CHIKV infected patients. While the frequency of IgM seroconversion among ZIKV infected patients was low (≈30%), CHIKV infected patients showed 100% of IgM seroconversion [55]. In addition, recent studies have reported extended duration of anti-CHIKV IgM detection for up to 18 months post-infection [13]. However, this represents only a relatively small fraction of CHIKV-infected patients (<8%) [56]. Thus, it is reasonable to speculate that the differences in the serological and molecular profile of CHIKV and ZIKV infections observed in our study might be related to the distinct patterns of viral RNA persistence, with longer persistence of ZIKV RNA among PW, and differences in IgM kinetics between these viruses. Finally, these results reinforce the extreme difficulty in establishing a definitive diagnosis of infection by ZIKV, even in symptomatic women, as demonstrated in an earlier study conducted in the same study setting [16].

In our study, less than 5% ZIKV or CHIKV infected PW were classified as suspected cases, according to WHO case definition [39,40] and around 80% did not report clinical symptoms of arbovirus disease during pregnancy. Clinical symptoms commonly associated with arbovirus diseases, such as fever or rash, were reported by 18.5% and 15.9% of ZIKV and CHIKV infected PW, respectively, similar frequency to those found in these recent studies with PW [11,57]. There were no differences in the frequency of symptoms reported by infected PW compared to uninfected PW, suggesting that most of these cases are not detected by surveillance systems and health care services. 

Similar results were observed in recent prospective studies that estimated ZIKV infection in PW in French Guiana [11] and Brazil [57], confirming the low accuracy of these clinical indices in the diagnosis of arbovirus-related obstetric complications in endemic areas.

Regarding obstetric complications, we highlight, in our study, that PW were recruited from a high-risk maternity hospital, with a high frequency of cases of hypertensive disorder of pregnancy and gestational diabetes, and others reported as obstetric complications, as expected. An interesting result is that the frequencies of these adverse events during pregnancy were similar between PW with active/recent ZIKV or CHIKV infection and uninfected PW. Our findings are in line with other cohort of ZIKV infection PW conducted in Rio de Janeiro [58], and another study conducted in Jundiaí, Sao Paulo [43], that could not differentiate a specific arbovirus-related obstetric complication in this population.

A limitation of our study is the absence of a comparison group of PW without obstetric complications, which would enable the analysis of the magnitude of association between arbovirus infection and obstetric complications. Nonetheless, our study provided relevant information about the burden of arboviruses infection among PW with complications potentially associated with maternal arbovirus infection during pregnancy in an area of high arbovirus transmission. Furthermore, our cohort differs from most previous reports of arbovirus-related complications in PW, which were restricted to those presenting symptomatic arbovirus disease, such as fever and/or rash. Our study enabled the characterization of the profile of laboratory markers of infection in paired samples, by combining multiple tests (qRT-PCR, ELISA, and PRNT). Finally, the persistence of ZIKV RNA detection during pregnancy and in the postpartum period reinforces the need for further studies to elucidate the mechanisms of prolonged ZIKV viremia in this population.

## 5. Conclusions

Our findings highlight the need for laboratory screening of arboviral infections in PW with complications throughout pregnancy and during childbirth, among those who reside in or travel to endemic areas. The creation of sentinel maternity hospitals, in order to monitor the arboviruses transmission and the adverse outcomes associated with these infections in PW and their infants, by clinical and laboratory screening, could be a useful measure to raise awareness on the increase in arboviral transmission and to guide decisions in clinical practice and public health, which can contribute for avoiding maternal and neonatal deaths. These findings should be considered when making future changes in the policies of epidemiological surveillance in PW. Finally, the development of highly accurate point-of-care tests that enable the early detection of arbovirus infection among PW with obstetric events, potentially associated with arboviral infections in high-risk pregnancy and childbirth care services from endemic areas, is important.

## Figures and Tables

**Table 1:** Sociodemographic and clinical characteristics and arbovirus immune status of the study population.

Characteristic	(*n* = 780) ^a^
*Sociodemographic*	
Age (in years), mean (±SD)	26.5 (3.6)
Self-reported Race/skin color, *n* (%)	
Mixed	480 (61.7)
White	157 (20.2)
Black	141 (18.1)
Family income (in minimum wages)	
≤2	640 (82.8)
>2	133 (17.2)
Schooling, *n* (%)	
Illiterate/Elementary	186 (23.8)
High school/Higher Education	594 (76.2)
*Clinical*	
Gestational age (in weeks) at admission, mean (±SD)	36.9 (±3.2)
Obstetric complication in previous pregnancy, *n* (%) ^b^	221 (45.9)
Prenatal consultation in the current pregnancy, *n (%)*	758 (97.2)
Pregnancy, *n* (%)	
Single	751(96.3)
Multiple	29 (3.7)
Type of obstetric complication, *n* (%)	
Hypertensive disorder of pregnancy ^c^	469 (60.1)
Gestational diabetes	98 (12.6)
Preterm labor/premature birth	110 (14.1)
HELLP syndrome (complete/incomplete)	35 (4.5)
Others ^d^	68 (8.7)
*Arbovirus infection status*, *n* (%)	
Previous DENV infection (IgG positives)	726 (93.1)
Active/recent DENV infection ^e^	3 (0.4)
Active/recent ZIKV infection ^e^	54 (6.9)
Active/recent CHIKV ^e^	69 (8.8)
Dual active/recent infection (CHIKV and ZIKV)	4 (0.5)

^a^ Numbers may vary due to missing values; ^b^ 481 multiparous pregnant women; ^c^ gestational hypertension, pre-eclampsia, or eclampsia; ^d^ obstetrical bleeding, oligohydramnios, chorioamnionitis, placenta abruption, and premature amniorrhexis. ^e^ qRT-PCR positive in the first sample and/or IgM or IgG (PRNT) seroconversion.

**Table 2:** Distribution of ZIKV and CHIKV mono and dual infection according to the case definition (active or recent) and laboratory assays.

Laboratorial Evidence	Arbovirus Infection			Total (*n* = 130) ^c^
	Mono-Infection		Dual-Infection	
	ZIKV (*n* = 54)	CHIKV (*n* = 69)	ZIKV + CHIKV (*n* = 4)	
*Case definition, n (%)*				
Active infection ^a^	49 (90.7)	30 (43.5)	3 (75.0)	84 (64.6)
Recent infection ^b^	5 (9.2)	39 (56.5)	1 (25.0)	46 (35.4)
*Laboratorial assays, n (%)*				
qRT-PCR positive (first sample)	48 (88.8)	2 (2.9)	-	-
Specific IgM positive (first sample)	5 (9.2)	39 (56.5)	-	-
qRT-PCR positive (first sample) and IgM seroconversion (paired samples)	1 (1.8)	0 (00)	-	-
IgM and/or IgG seroconversion (paired samples)	0 (0.0)	28 (40.6)	-	-
ZIKV and CHIKV qRT-PCR positives (First sample)	-	-	1 (25.0)	-
ZIKV qRT-PCR positive and CHIKV IgM seroconversion	-	-	2 (50.0)	-
ZIKV and CHIKV IgM positive (first sample)	-	-	1 (25.0)	-

^a^ qRT-PCR positive in the first sample and/or IgM or IgG seroconversion (PRNT); ^b^ qRT-PCR negative and IgM positive in the first or paired samples; ^c^ including three cases of DENV mono-infection.

**Table 3:** Characteristics of pregnant women with active/recent ZIKV or CHIKV infection and those uninfected according to the reported symptoms and obstetric complications in a high-risk maternity hospital in Northeastern Brazil.

Clinical Characteristics	ZIKV *(n* = 54)	CHIKV (*n* = 69)	Uninfected *(n* = 650)	*p* Value ^a^
	*n* (%)	*n* (%)	*n* (%)	
*Self-Reported Signs and Symptoms*				
Suspected cases (WHO case definition) ^b^	2 (3.7)	3 (4.3)	32 (4.9)	0.914
Acute febrile episodes	7 (12.9)	8 (11.6)	111 (17.1)	0.503
Rash	3 (5.5)	6 (8.7)	31 (4.8)	0.420
Arthralgia	4 (7.4)	13 (18.8)	97 (14.9)	0.285
Fever and/or rash	10 (18.5)	11 (15.9)	129 (19.8)	0.802
Zika, Dengue, or Chikungunya in current pregnancy	0 (0.0)	2 (3.1)	2 (0.3)	-
*Obstetrical complications* ^c^				
Hypertensive disorder of pregnancy	30 (57.7)	42 (60.9)	390 (60.0)	0.983
Gestational diabetes	8 (15.4)	7 (10.1)	82 (12.6)	0.749
Preterm labor/premature birth	7 (13.4)	11 (15.9)	91 (14.0)	0.792
HELLP syndrome (complete/incomplete)	3 (5.8)	4 (5.8)	28 (4.3)	0.792
Others	4 (7.7)	5 (7.2)	59 (9.1)	0.862

^a^ Chi-square test (2 degree of freedom); ^b^ case definition: Zika suspected case WHO, 2016 [39]: rash and/or fever and at least one of these signs or symptoms: arthralgia, arthritis, or conjunctivitis. Chikungunya suspected case [40]: triad of fever, rash, and joint manifestations. ^c^ Four PW with ZIKV and CHIKV co-infection and two with active ZIKV infection after delivery were excluded.

## Data Availability

The data presented in this study are available on request from the corresponding author.
